# Sexually Dimorphic Formation of the Preoptic Area and the Bed Nucleus of the Stria Terminalis by Neuroestrogens

**DOI:** 10.3389/fnins.2020.00797

**Published:** 2020-07-29

**Authors:** Shinji Tsukahara, Masahiro Morishita

**Affiliations:** Division of Life Science, Graduate School of Science and Engineering, Saitama University, Saitama, Japan

**Keywords:** sexual differentiation of the brain, sexually dimorphic nucleus, sex difference, androgens, estrogens, preoptic area, bed nucleus of the stria terminalis

## Abstract

Testicular androgens during the perinatal period play an important role in the sexual differentiation of the brain of rodents. Testicular androgens transported into the brain act via androgen receptors or are the substrate of aromatase, which synthesizes neuroestrogens that act via estrogen receptors. The latter that occurs in the perinatal period significantly contributes to the sexual differentiation of the brain. The preoptic area (POA) and the bed nucleus of the stria terminalis (BNST) are sexually dimorphic brain regions that are involved in the regulation of sex-specific social behaviors and the reproductive neuroendocrine system. Here, we discuss how neuroestrogens of testicular origin act in the perinatal period to organize the sexually dimorphic structures of the POA and BNST. Accumulating data from rodent studies suggest that neuroestrogens induce the sex differences in glial and immune cells, which play an important role in the sexually dimorphic formation of the dendritic synapse patterning in the POA, and induce the sex differences in the cell number of specific neuronal cell groups in the POA and BNST, which may be established by controlling the number of cells dying by apoptosis or the phenotypic organization of living cells. Testicular androgens in the peripubertal period also contribute to the sexual differentiation of the POA and BNST, and thus their aromatization to estrogens may be unnecessary. Additionally, we discuss the notion that testicular androgens that do not aromatize to estrogens can also induce significant effects on the sexually dimorphic formation of the POA and BNST.

## Introduction

Sex differences in the structures of the brain are considered to underlie sex-specific functions of the brain and brain functions that differ between sexes or genders. The mechanisms by which the brain is sexually differentiated have not yet been completely elucidated; however, they have long been studied using animal models, especially rodents. Based on accumulated data, androgens secreted from the testes during the perinatal period are converted to estrogens in the brain, wherein the neuroestrogens masculinize and defeminize the brain. Neuroestrogens are essential but not sole factors in the sexual differentiation of the brain. There are other factors that significantly contribute to the brain sexual differentiation. The processes of brain sexual differentiation require sex chromosome genes’ expression in the brain (McCarthy and Arnold, 2011; Cox et al., 2014) and gonadal steroids secreted during the peripubertal period (Juraska et al., 2013; Schulz and Sisk, 2016). However, there is no doubt that neuroestrogens of testicular origin play an important role in the sexual differentiation of the brain. In this mini review, we focused on two sexually dimorphic brain regions: the preoptic area (POA) and the bed nucleus of the stria terminalis (BNST), which are involved in the regulation of sexually dimorphic social behaviors and reproductive neuroendocrine functions. First, we give an overview of the sex differences in the POA and BNST of the rodent brain. Second, we discuss how neuroestrogens masculinize or defeminize the POA and BNST. Third, we further discuss the notion that testicular androgens that do not aromatize into estrogens can also induce the sexually dimorphic formation of the POA and BNST.

## Sex Differences in the POA and BNST

**FIGURE 1:**
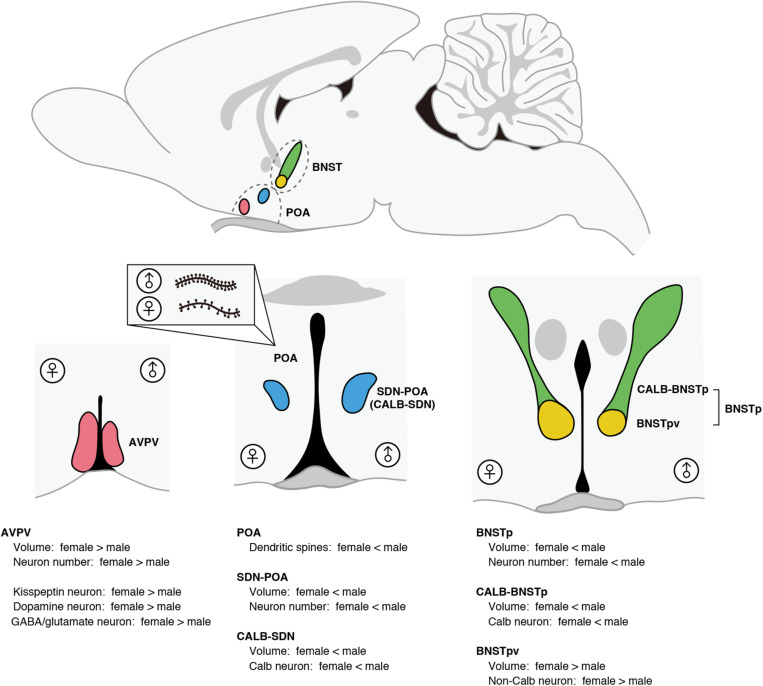
Morphological sex differences in the POA and BNST of rats and mice. The AVPV exhibits female-biased sex differences in volume and neuron number. Compared with males, females have a greater number of kisspeptin neurons, dopamine neurons, and GABA/glutamate neurons in the AVPV. The SDN-POA exhibits male-biased sex differences in volume and neuron number. The SDN-POA contains a cluster of Calb neurons termed CALB-SDN. The CALB-SDN is larger in volume and contains a larger number of Calb neurons in males than in females. The POA shows a sex difference in the dendritic synapse pattern. POA neurons in males have more dendritic spines compared with POA neurons in females. The BNST contains a male-biased sexually dimorphic nucleus (BNSTp), which is composed of a sexually dimorphic subregion that is larger in volume and number of Calb neurons in males (CALB-BNSTp) and a sexually dimorphic subregion that is larger in volume and number of non-Calb neurons in females (BNSTpv).

The POA and BNST show morphological sex differences that are related to sex-specific brain functions (Figure 1). The number of dendritic spine synapses in the POA is twofold greater in male rats than in females; the POA masculinized by neuroestrogens, resulting in a greater number of dendritic spine synapses, plays an important role in the control of male sexual behavior (Amateau and McCarthy, 2002a, 2004; Wright et al., 2008; Wright and McCarthy, 2009). The male-biased sex difference in dendritic spine synapses in the POA is established by the crosstalk between neuroendocrine and immune systems where microglia and mast cells have significant roles [see reviews (Arambula and McCarthy, 2020; McCarthy, 2020) and the next section].

In the POA of rats and mice, there are two sexually dimorphic nuclei that have been identified to date. The sexually dimorphic nucleus of the POA (SDN-POA) exhibits male-biased sex differences in volume and the number of neurons (Gorski et al., 1978, 1980). The SDN-POA of male rats has been suggested to be related to partner preference (Houtsmuller et al., 1994; Woodson et al., 2002) and sexual arousal (Arendash and Gorski, 1983; De Jonge et al., 1989; Maejima et al., 2018); however, the physiological functions of the SDN-POA require further investigation. Approximately half of the SDN-POA neurons express calbindin-D28K (Calb) (Morishita et al., 2017), a calcium-binding protein that functions as a buffer, sensor, and transporter of calcium (Schmidt, 2012). A cluster of Calb neurons in the SDN-POA is termed the calbindin-sexually dimorphic nucleus (CALB-SDN), which has more Calb neurons in males than in females (Sickel and McCarthy, 2000; Edelmann et al., 2007; Orikasa and Sakuma, 2010). Although the physiological roles of Calb neurons remain unclear, Calb neurons in male rats are activated during sexual behavior (Yamaguchi et al., 2018).

Another sexually dimorphic nucleus in the POA is the anteroventral periventricular nucleus (AVPV), which is larger and contains more neurons in females than in males (Bleier et al., 1982). The AVPV of rats contains neurons expressing kisspeptin, neurons producing dopamine, and neurons producing both GABA and glutamate, and exhibits a female-biased sex difference in the number of these neurons (Simerly et al., 1985a; Ottem et al., 2004; Kauffman et al., 2007). In the AVPV of mice, approximately half of kisspeptin neurons produce dopamine and vice versa (Clarkson and Herbison, 2011). In the female AVPV, kisspeptin neurons expressing estrogen receptor α (ERα) are a target of the positive feedback actions of ovarian estrogens to induce ovulation (Kauffman, 2009; Tsukamura et al., 2010). Furthermore, kisspeptin neurons in the AVPV of female mice are key players in orchestrating successful reproduction by synchronizing copulation with ovulation (Hellier et al., 2018). Dual-phenotype GABA/glutamate neurons in the AVPV of rats and mice interact with gonadotropin-releasing hormone neurons to excite or inhibit their activity (Ottem et al., 2004; Liu et al., 2011). Dopamine neurons in the AVPV of female mice enhance maternal behavior, whereas dopamine neurons in the AVPV of male mice do not affect parental behavior, but suppress intermale aggression (Scott et al., 2015).

The principal nucleus of the BNST (BNSTp) is a subnucleus of the BNST showing male-biased sex differences in size and neuron number (Hines et al., 1985, 1992). Like the SDN-POA, the BNSTp contains more Calb neurons in male mice than in female mice (Gilmore et al., 2012). The subregion of the BNSTp that contains many Calb neurons and exhibits the male-biased sex difference in the number of Calb neurons is hereinafter referred to as CALB-BNSTp. BNSTp neurons expressing aromatase in male mice are necessary to distinguish the conspecific sexes and ensure social interactions (Bayless et al., 2019). However, the physiological functions of Calb neurons in the BNSTp remain unclear. Unlike the CALB-BNSTp, the ventral part of the BNSTp (BNSTpv) contains few Calb neurons without sex differences, but the BNSTpv is larger and has more non-Calb neurons in female mice than in males (Moe et al., 2016; Morishita et al., 2017). Thus, the BNSTp is composed of a region exhibiting male-biased sex differences in Calb neurons and a region exhibiting female-biased sex differences in non-Calb neurons.

## Neuroestrogens of Testicular Origin are Significant Factors for Sexually Dimorphic Formation of the POA and BNST

**FIGURE 2:**
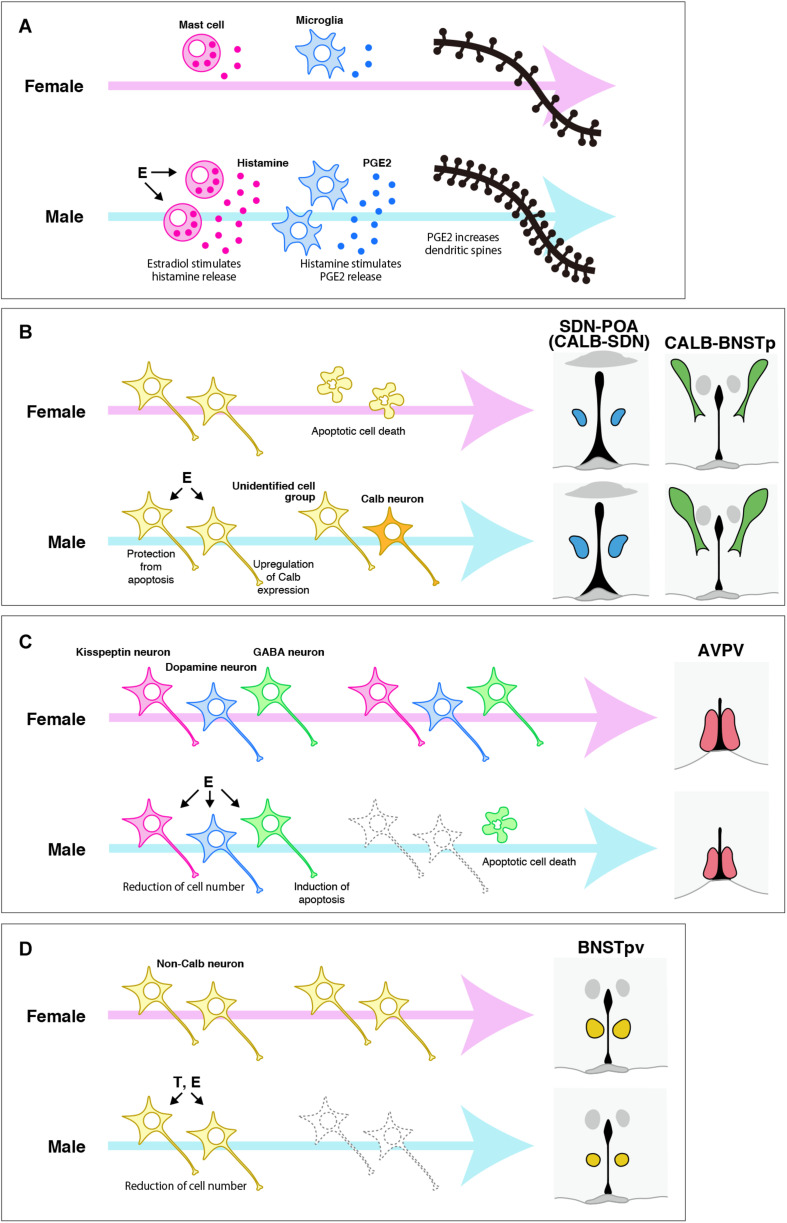
Possible mechanisms for organizing the sexually dimorphic structures of the POA and BNST of rats and mice. **(A)** The mechanism responsible for the sexual differentiation of dendritic synapse patterning in the POA, which was proposed by McCarthy and colleagues (see reviews Arambula and McCarthy, 2020; McCarthy, 2020). Estradiol (E), which is synthetized in the brain from testicular testosterone (T) during the perinatal period, stimulates histamine release from mast cells, which then stimulates prostaglandin E2 (PGE2) to increase dendritic spines. **(B)** The mechanism for organizing the sexually dimorphic structures of the SDN-POA/CALB-SDN and CALB-BNSTp. E originating from testicular T in the perinatal period protects a population of neurons from apoptotic cell death, although the neurochemical properties of the cell population have not been identified. Additionally, E may upregulate Calb expression, followed by induction of a male-biased sex difference in the number of Calb neurons. **(C)** The mechanisms for inducing a female-biased sex difference in the number of cells in the specific neuronal cell groups in the AVPV. E originating from testicular T in the perinatal period induces the death of GABA neurons by apoptosis to reduce their number. E may also reduce the number of kisspeptin neurons and dopamine neurons by a mechanism other than apoptosis, although the mechanism remains unknown. **(D)** The roles of T and E during the perinatal period in the sexual differentiation of the BNSTpv. T and E reduce the number of BNSTpv neurons that do not express Calb. However, identification of the neurochemical properties and the mechanism responsible for inducing the sex difference require further investigation.

Neuroestrogens originating from testicular androgens affect the POA and BNST in the perinatal period to organize sexually dimorphic structures in a variety of modes of action (Figure 2). As mentioned before, the POA of rats has more dendritic spines in males than in females. The increased number of dendritic spines in the male POA is induced by estrogens in the perinatal period (Amateau and McCarthy, 2002a, 2004). The mechanisms responsible for the masculinization of dendritic spine patterning by estrogens are considered to be as follows. First, neuroestrogens originating from testicular androgens during the perinatal period affect mast cells in the POA via ER to stimulate histamine release, which then stimulates microglia in the POA to release prostaglandin E2, which triggers POA neurons to increase dendritic spine synapses via induction of glutamate receptor signaling (Wright et al., 2008; Wright and McCarthy, 2009; Lenz et al., 2011, 2013, 2018). Thus, microglia and mast cells have critical roles in the masculinization of dendritic spine patterning. The POA of postnatal males has twice as many ameboid microglia, a class of microglia with a more activated morphological profile, compared with postnatal females (Lenz et al., 2013). The male-biased sex difference in ameboid microglia is regulated by neuroestrogens of testicular origin, because treatment with estradiol increased ameboid microglia in the POA of postnatal females (Lenz et al., 2013). The male POA has more activated mast cells than the female POA in the perinatal period, and approximately half of the mast cells in both sexes express ERα (Lenz et al., 2018). Additionally, astrocytes in the POA of postnatal rats exhibit a sex difference in morphology: astrocytes in males have longer and more primary processes, and the astrocyte morphology is masculinized by neuroestrogens in the postnatal period (Amateau and McCarthy, 2002b). Astrocytes release chemical transmitters, including glutamate, and are involved in synapse formation (Araque et al., 1999). Taken together, the sexually dimorphic synapse formation may follow the sexual differentiation of these non-neuronal cells.

The masculinization of the SDN-POA/CALB-SDN requires the actions of estrogens that are synthesized in the brain from testicular androgens and act via ERα during the postnatal period (Gorski et al., 1978; Patchev et al., 2004; Orikasa and Sakuma, 2010; Gilmore et al., 2012; Morishita et al., 2017). How neuroestrogens masculinize the SDN-POA/CALB-SDN is not completely understood. However, controlling cell numbers by apoptosis during the postnatal period appears to produce a male-biased sex difference in the number of SDN-POA neurons. The number of cells generated during the late fetal period and incorporated into the SDN-POA during the neonatal period does not differ between sexes (Jacobson et al., 1985; Dodson et al., 1988; Kato et al., 2012), but the number of apoptotic cells in the SDN-POA of postnatal rats is smaller in males because of suppression of apoptosis by neuroestrogens of testicular origin (Arai et al., 1996; Davis et al., 1996; Chung et al., 2000). Postnatal apoptosis in the SDN-POA is regulated by the mitochondrial apoptotic pathway involving Bcl-2, Bax, and caspase-3. In the SDN-POA of postnatal rats, the expression of Bcl-2 and Bax is higher in males and in females, respectively, followed by higher activity of caspoase-3 in males (Tsukahara et al., 2006). The sex differences in Bcl-2 and Bax expression result from the upregulation of Bcl-2 expression and downregulation of Bax expression by neuroestrogens of testicular origin because estradiol treatment increased the Bcl-2 protein level and decreased the Bax protein level in the POA of postnatal female rats (Tsukahara et al., 2008). Nevertheless, the sex difference in the number of Calb neurons may occur independently of apoptotic regulation, because deletion of the *Bax* gene did not affect the number of Calb neurons in the CALB-SDN of mice in both sexes (Gilmore et al., 2012). Calb expression may be upregulated by neuroestrogens of testicular origin; the number of Calb neurons is increased in male mice, because the mouse *Calb* promoter possesses estrogen-responsive elements and is estrogen responsive (Gill and Christakos, 1995). Ca^2+^ is a key regulator of cellular functions in living cells, but it also induces apoptosis upon prolonged changes in its intercellular concentrations, including an increase in its cytoplasmic and mitochondrial concentrations (Hajnoczky et al., 2003). Calb protects neurons from cell death by chelating intercellular Ca^2+^ (Meier et al., 1998; D’Orlando et al., 2002; Fan et al., 2007). It also prevents neuronal cell death by inhibiting caspase-3 activity (Choi et al., 2008; Choi and Oh, 2014). These findings may support the notion that Calb upregulation by estrogens prevents apoptotic cell death.

Neuroestrogens originating from testicular androgens in the perinatal period reduce the total number of neurons to defeminize the AVPV in rats and mice (Patchev et al., 2004; Kanaya et al., 2014). Furthermore, these neuroestrogens defeminized specific neuronal cell groups in the AVPV by reducing their cell number. The number of kisspeptin neurons in the AVPV increased in male rats with neonatal castration and decreased in female rats with neonatal estradiol treatment (Kauffman et al., 2007; Homma et al., 2009). Perinatal or neonatal testosterone treatment reduced the number of dopamine neurons in the AVPV of female rats (Simerly et al., 1985b). The number of dopamine neurons in the AVPV of male mice increased upon the deletion of the genes for ERα and ERβ, resulting in the disappearance of the sex difference in dopamine neurons (Simerly et al., 1997; Bodo et al., 2006). Male rodents have a greater number of apoptotic cells in the AVPV during the perinatal period than female rodents do, which is attributed to the induction of apoptosis by estrogens (Sumida et al., 1993; Arai et al., 1996; Yoshida et al., 2000; Waters and Simerly, 2009). Controlling the number of neurons by apoptosis via Bcl-2 and Bax is required for the sexually dimorphic formation of the AVPV of mice (Zup et al., 2003; Forger et al., 2004). The AVPV of postnatal rats shows a male-biased sex difference in Bax expression and a female-biased sex difference in Bcl-2 expression, followed by higher activity of caspase-3 in the male AVPV (Tsukahara et al., 2006). In addition, the tumor necrosis factor α (TNF-α)-TNF receptor 2 (TNFR2)-NFκB cell survival pathway is activated in the AVPV of postnatal female rats to upregulate Bcl-2 expression, whereas this pathway is suppressed by TNF receptor-associated factor 2-inhibitng protein (TRIP) in the male AVPV, followed by an increase in the number of apoptotic cells (Krishnan et al., 2009). Postnatal apoptosis regulated by this pathway may result in a sex difference in GABA neurons of the AVPV (Krishnan et al., 2009). However, the sex differences in the number of dopamine and kisspeptin neurons in the AVPV of mice are independent of Bcl-2 and Bax (Zup et al., 2003; Forger et al., 2004; Semaan et al., 2010). There may be other mechanisms that establish the sex differences in dopamine and kisspeptin neurons, although they remain nuclear.

Estrogens that are synthesized in the brain from testicular androgens and act via ERα during the postnatal period masculinize the BNSTp by increasing the volume and neuron number in rats and mice (Guillamon et al., 1988; Chung et al., 2000; Hisasue et al., 2010; Tsukahara et al., 2011). The number of apoptotic cells in the BNSTp of postnatal rats is smaller in males because of the protection of cells from apoptosis by neonatal testicular androgens (Chung et al., 2000). This indicates that suppression of apoptotic cell death by testicular androgens contributes to the masculinization of the BNSTp. The apoptotic pathway involving Bax accounts for the male-biased sex difference in the number of BNSTp neurons in adulthood following the female-biased sex difference in postnatal apoptosis (Forger et al., 2004; Gotsiridze et al., 2007). Like the BNSTp, the CALB-BNSTp in mice is masculinized by postnatal testicular androgens, which act after aromatization (Morishita et al., 2017). However, Bax-dependent apoptosis may not be necessary for establishing the sex difference in the number of Calb neurons, because deletion of the *Bax* gene increased Calb neurons in both sexes, but did not eliminate the sex difference (Gilmore et al., 2012). As mentioned earlier, estradiol can induce Calb expression (Gill and Christakos, 1995). Phenotypic organization induced by estrogens is a possible mechanism for the sexual differentiation of Calb neurons, although this idea needs to be investigated. The volume and the number of non-CALB neurons in the BNSTpv increased in male mice with neonatal castration and decreased in female mice upon postnatal treatment with estradiol or dihydrotestosterone (Morishita et al., 2017), suggesting that testicular androgens affect the BNSTpv after aromatizing to estrogens, but they also affect this area without aromatization.

In rodents, the critical time window in which neuroestrogens effectively induce brain sexual differentiation is limited to the perinatal period. Nevertheless, the effects of neuroestrogens persist until adulthood. The long-lasting effects are considered to be due to epigenetic changes in gene expression [see reviews (Forger, 2016, 2018; McCarthy, 2019)]. In fact, some of the aforementioned sex differences emerge via epigenetic regulation. Compared with postnatal males, postnatal female rats have higher DNA methyltransferase activity in the POA, which is followed by higher DNA methylation, and postnatal estradiol treatment reduced DNA methyltransferase activity and DNA methylation in the female POA (Nugent et al., 2015). Moreover, inhibition of DNA methyltransferase in the brain of neonatal females increased dendritic spines of POA neurons and masculinized sexual behavior (Nugent et al., 2015), and increased the number of Calb neurons in the CALB-SDN and CALB-BNSTp, resulting in elimination of the sex difference in Calb neurons (Mosley et al., 2017; Cisternas et al., 2020). Epigenetic regulation via histone modification also contributes to masculinization of the brain. Inhibition of histone deacetylase in the brain reduced the number of BNSTp neurons in male mice and neonatally testosterone-treated females (Murray et al., 2009) and reduced the activity of sexual behavior in male rats (Matsuda et al., 2011). ER is a ligand-activated transcription factor, and thereby estrogens binding to ER modulate the expression of the target genes at the transcriptional level. Therefore, though it may not be the whole story, epigenetic regulation by estrogens is an essential part of the molecular mechanisms of brain sexual differentiation.

## Sexually Dimorphic Formation of the POA and BNST Requires Neuroestrogens of Testicular Origin and Testicular Androgens

Masculinization of the BNSTp is disrupted in rats with reduced functional androgen receptors (ARs) (Durazzo et al., 2007) and AR-knockout mice (Kanaya et al., 2014), indicating that the masculinization of the BNSTp requires the actions of testicular androgens via AR. The BNSTp of mice begins to express AR from the neonatal period, but the expression level is low until one week after birth (Juntti et al., 2010; Kanaya et al., 2014). It seems likely that the androgen actions via AR mainly occur after the perinatal period. The sex difference in the number of Calb neurons in the CALB-SDN and CALB-BNSTp of mice emerges before puberty and becomes pronounced after puberty (Wittmann and McLennan, 2013a, b; Morishita et al., 2017). This is partly due to an increase in the number of Calb neurons during the peripubertal period, which is induced by testicular androgens via AR because the decrease in the number of Calb neurons by prepubertal castration was reversed by peripubertal treatment with dihydrotestosterone, but not with estradiol (Morishita et al., 2020). Thus, testicular androgens that are synthesized during the peripubertal period and act via ARs are necessary for the masculinization of Calb neurons. However, it cannot be excluded that masculinization of the brain requires the actions of neuroestrogens during puberty, because prepubertal knockdown of ERα in the medial amygdala, a male-biased sexually dimorphic nucleus, disrupts the masculinization of this nucleus in mice (Sano et al., 2016).

## Conclusion

Perinatal testicular androgens induce masculinizing and defeminizing effects on the POA and BNST of rodents through binding to ER after conversion to estrogens in the brain rather than by binding to AR directly, resulting in sex differences in glial and immune cells, dendritic synapse patterning, and specific neuronal cell groups. Interactions among immune cells, glial cells, and neuronal cells under the influence of neuroestrogens is a prerequisite for producing the sex difference in dendritic synapse patterning in the POA. Sex differences in specific neuronal cell groups in the SDN-POA, AVPV, and BNSTp may be established by controlling the number of dying cells by apoptosis or phenotypic organization of living cells that are influenced by neuroestrogens. Neuroestrogens binding to ER modulate the expression of the target genes at the transcriptional level, but also modulate gene expression by epigenetic regulation, which ensures the long-lasting effects of neuroestrogens beyond the perinatal period. Testicular androgens in the peripubertal period also contribute to the sexual differentiation of the POA and BNST, but aromatizing them to estrogens may not be necessary. Thus, peripubertal testicular androgens can act via AR directly to masculinize the sexually dimorphic nuclei.

## Author Contributions

MM and ST prepared the manuscript. Both authors contributed to the article and approved the submitted version.

## Conflict of Interest

The authors declare that the research was conducted in the absence of any commercial or financial relationships that could be construed as a potential conflict of interest.

## References

[B1] Amateau S. K. McCarthy M. M. (2002a). A novel mechanism of dendritic spine plasticity involving estradiol induction of prostaglandin-E2. *J. Neurosci.* 22 8586–8596. 10.1523/jneurosci.22-19-08586.2002 12351732 PMC6757802

[B2] Amateau S. K. McCarthy M. M. (2002b). Sexual differentiation of astrocyte morphology in the developing rat preoptic area. *J. Neuroendocrinol.* 14 904–910. 10.1046/j.1365-2826.2002.00858.x 12421344

[B3] Amateau S. K. McCarthy M. M. (2004). Induction of PGE2 by estradiol mediates developmental masculinization of sex behavior. *Nat. Neurosci.* 7 643–650. 10.1038/nn1254 15156148

[B4] Arai Y. Sekine Y. Murakami S. (1996). Estrogen and apoptosis in the developing sexually dimorphic preoptic area in female rats. *Neurosci. Res.* 25 403–407. 10.1016/0168-0102(96)01070-x 8866522

[B5] Arambula S. E. McCarthy M. M. (2020). Neuroendocrine-immune crosstalk shapes sex-specific brain development. *Endocrinology* 161:bqaa055. 10.1210/endocr/bqaa055 32270188 PMC7217281

[B6] Araque A. Parpura V. Sanzgiri R. P. Haydon P. G. (1999). Tripartite synapses: glia, the unacknowledged partner. *Trends Neurosci.* 22 208–215. 10.1016/s0166-2236(98)01349-6 10322493

[B7] Arendash G. W. Gorski R. A. (1983). Effects of discrete lesions of the sexually dimorphic nucleus of the preoptic area or other medial preoptic regions on the sexual behavior of male rats. *Brain Res. Bull.* 10 147–154. 10.1016/0361-9230(83)90086-2 6824962

[B8] Bayless D. W. Yang T. Mason M. M. Susanto A. A. T. Lobdell A. Shah N. M. (2019). Limbic neurons shape sex recognition and social behavior in sexually naive males. *Cell* 176 1190–1205.e20. 10.1016/j.cell.2018.12.041 30712868 PMC6453703

[B9] Bleier R. Byne W. Siggelkow I. (1982). Cytoarchitectonic sexual dimorphisms of the medial preoptic and anterior hypothalamic areas in guinea pig, rat, hamster, and mouse. *J. Comp. Neurol.* 212 118–130. 10.1002/cne.902120203 7187914

[B10] Bodo C. Kudwa A. E. Rissman E. F. (2006). Both estrogen receptor-alpha and -beta are required for sexual differentiation of the anteroventral periventricular area in mice. *Endocrinology* 147 415–420. 10.1210/en.2005-0834 16239299

[B11] Choi W. S. Lee E. Lim J. Oh Y. J. (2008). Calbindin-D28K prevents drug-induced dopaminergic neuronal death by inhibiting caspase and calpain activity. *Biochem. Biophys. Res. Commun.* 371 127–131. 10.1016/j.bbrc.2008.04.020 18413141

[B12] Choi W. S. Oh Y. J. (2014). Calbindin-D28K Prevents Staurosporin-induced Bax Cleavage and Membrane Permeabilization. *Exp. Neurobiol.* 23 173–177. 10.5607/en.2014.23.2.173 24963283 PMC4065832

[B13] Chung W. C. Swaab D. F. De Vries G. J. (2000). Apoptosis during sexual differentiation of the bed nucleus of the stria terminalis in the rat brain. *J. Neurobiol.* 43 234–243. 10.1002/(sici)1097-4695(20000605)43:3<234::aid-neu2>3.0.co;2-3 10842236

[B14] Cisternas C. D. Cortes L. R. Golynker I. Castillo-Ruiz A. Forger N. G. (2020). Neonatal inhibition of DNA methylation disrupts testosterone-dependent masculinization of neurochemical phenotype. *Endocrinology* 161:bqz022. 10.1210/endocr/bqz022 31742329

[B15] Clarkson J. Herbison A. E. (2011). Dual phenotype kisspeptin-dopamine neurones of the rostral periventricular area of the third ventricle project to gonadotrophin-releasing hormone neurones. *J. Neuroendocrinol.* 23 293–301. 10.1111/j.1365-2826.2011.02107.x 21219482

[B16] Cox K. H. Bonthuis P. J. Rissman E. F. (2014). Mouse model systems to study sex chromosome genes and behavior: relevance to humans. *Front. Neuroendocrinol.* 35 405–419. 10.1016/j.yfrne.2013.12.004 24388960 PMC4079771

[B17] Davis E. C. Popper P. Gorski R. A. (1996). The role of apoptosis in sexual differentiation of the rat sexually dimorphic nucleus of the preoptic area. *Brain Res.* 734 10–18. 10.1016/0006-8993(96)00298-3 8896803

[B18] De Jonge F. H. Louwerse A. L. Ooms M. P. Evers P. Endert E. van de Poll N. E. (1989). Lesions of the SDN-POA inhibit sexual behavior of male Wistar rats. *Brain Res. Bull.* 23 483–492. 10.1016/0361-9230(89)90194-9 2611691

[B19] Dodson R. E. Shryne J. E. Gorski R. A. (1988). Hormonal modification of the number of total and late-arising neurons in the central part of the medial preoptic nucleus of the rat. *J. Comp. Neurol.* 275 623–629. 10.1002/cne.902750410 3192761

[B20] D’Orlando C. Celio M. R. Schwaller B. (2002). Calretinin and calbindin D-28k, but not parvalbumin protect against glutamate-induced delayed excitotoxicity in transfected N18-RE 105 neuroblastoma-retina hybrid cells. *Brain Res.* 945 181–190. 10.1016/s0006-8993(02)02753-1 12126880

[B21] Durazzo A. Morris J. A. Breedlove S. M. Jordan C. L. (2007). Effects of the testicular feminization mutation (tfm) of the androgen receptor gene on BSTMPM volume and morphology in rats. *Neurosci. Lett.* 419 168–171. 10.1016/j.neulet.2007.04.033 17490813

[B22] Edelmann M. Wolfe C. Scordalakes E. M. Rissman E. F. Tobet S. (2007). Neuronal nitric oxide synthase and calbindin delineate sex differences in the developing hypothalamus and preoptic area. *Dev. Neurobiol.* 67 1371–1381. 10.1002/dneu.20507 17638388 PMC3622702

[B23] Fan Y. Shi L. Gu Y. Zhao Y. Xie J. Qiao J. (2007). Pretreatment with PTD-calbindin D 28k alleviates rat brain injury induced by ischemia and reperfusion. *J. Cereb. Blood Flow Metab.* 27 719–728. 10.1038/sj.jcbfm.9600373 16868556

[B24] Forger N. G. (2016). Epigenetic mechanisms in sexual differentiation of the brain and behaviour. *Philos. Trans. R. Soc. Lond. B Biol. Sci.* 371:20150114. 10.1098/rstb.2015.0114 26833835 PMC4785900

[B25] Forger N. G. (2018). Past, present and future of epigenetics in brain sexual differentiation. *J. Neuroendocrinol.* 30:e12492. 10.1111/jne.12492 28585265

[B26] Forger N. G. Rosen G. J. Waters E. M. Jacob D. Simerly R. B. de Vries G. J. (2004). Deletion of Bax eliminates sex differences in the mouse forebrain. *Proc. Natl. Acad. Sci. U.S.A.* 101 13666–13671. 10.1073/pnas.0404644101 15342910 PMC518810

[B27] Gill R. K. Christakos S. (1995). Regulation by estrogen through the 5’-flanking region of the mouse calbindin-D28k gene. *Mol. Endocrinol.* 9 319–326. 10.1210/mend.9.3.7776978 7776978

[B28] Gilmore R. F. Varnum M. M. Forger N. G. (2012). Effects of blocking developmental cell death on sexually dimorphic calbindin cell groups in the preoptic area and bed nucleus of the stria terminalis. *Biol. Sex Differ.* 3:5. 10.1186/2042-6410-3-5 22336348 PMC3305593

[B29] Gorski R. A. Gordon J. H. Shryne J. E. Southam A. M. (1978). Evidence for a morphological sex difference within the medial preoptic area of the rat brain. *Brain Res.* 148 333–346. 10.1016/0006-8993(78)90723-0 656937

[B30] Gorski R. A. Harlan R. E. Jacobson C. D. Shryne J. E. Southam A. M. (1980). Evidence for the existence of a sexually dimorphic nucleus in the preoptic area of the rat. *J. Comp. Neurol.* 193 529–539. 10.1002/cne.901930214 7440781

[B31] Gotsiridze T. Kang N. Jacob D. Forger N. G. (2007). Development of sex differences in the principal nucleus of the bed nucleus of the stria terminalis of mice: role of Bax-dependent cell death. *Dev. Neurobiol.* 67 355–362. 10.1002/dneu.20353 17443793

[B32] Guillamon A. Segovia S. del Abril A. (1988). Early effects of gonadal steroids on the neuron number in the medial posterior region and the lateral division of the bed nucleus of the stria terminalis in the rat. *Brain Res. Dev. Brain Res.* 44 281–290. 10.1016/0165-3806(88)90226-x 3224428

[B33] Hajnoczky G. Davies E. Madesh M. (2003). Calcium signaling and apoptosis. *Biochem. Biophys. Res. Commun.* 304 445–454. 10.1016/s0006-291x(03)00616-8 12729578

[B34] Hellier V. Brock O. Candlish M. Desroziers E. Aoki M. Mayer C. (2018). Female sexual behavior in mice is controlled by kisspeptin neurons. *Nat. Commun.* 9:400. 10.1038/s41467-017-02797-2 29374161 PMC5786055

[B35] Hines M. Allen L. S. Gorski R. A. (1992). Sex differences in subregions of the medial nucleus of the amygdala and the bed nucleus of the stria terminalis of the rat. *Brain Res.* 579 321–326. 10.1016/0006-8993(92)90068-k 1352729

[B36] Hines M. Davis F. C. Coquelin A. Goy R. W. Gorski R. A. (1985). Sexually dimorphic regions in the medial preoptic area and the bed nucleus of the stria terminalis of the guinea pig brain: a description and an investigation of their relationship to gonadal steroids in adulthood. *J. Neurosci.* 5 40–47. 10.1523/jneurosci.05-01-00040.1985 3965644 PMC6565092

[B37] Hisasue S. Seney M. L. Immerman E. Forger N. G. (2010). Control of cell number in the bed nucleus of the stria terminalis of mice: role of testosterone metabolites and estrogen receptor subtypes. *J. Sex Med.* 7 1401–1409. 10.1111/j.1743-6109.2009.01669.x 20102443

[B38] Homma T. Sakakibara M. Yamada S. Kinoshita M. Iwata K. Tomikawa J. (2009). Significance of neonatal testicular sex steroids to defeminize anteroventral periventricular kisspeptin neurons and the GnRH/LH surge system in male rats. *Biol. Reprod.* 81 1216–1225. 10.1095/biolreprod.109.078311 19684332

[B39] Houtsmuller E. J. Brand T. de Jonge F. H. Joosten R. N. van de Poll N. E. Slob A. K. (1994). SDN-POA volume, sexual behavior, and partner preference of male rats affected by perinatal treatment with ATD. *Physiol. Behav.* 56 535–541. 10.1016/0031-9384(94)90298-4 7972405

[B40] Jacobson C. D. Davis F. C. Gorski R. A. (1985). Formation of the sexually dimorphic nucleus of the preoptic area: neuronal growth, migration and changes in cell number. *Brain Res.* 353 7–18. 10.1016/0165-3806(85)90019-7 4027683

[B41] Juntti S. A. Tollkuhn J. Wu M. V. Fraser E. J. Soderborg T. Tan S. (2010). The androgen receptor governs the execution, but not programming, of male sexual and territorial behaviors. *Neuron* 66 260–272. 10.1016/j.neuron.2010.03.024 20435002 PMC2923659

[B42] Juraska J. M. Sisk C. L. DonCarlos L. L. (2013). Sexual differentiation of the adolescent rodent brain: hormonal influences and developmental mechanisms. *Horm. Behav.* 64 203–210. 10.1016/j.yhbeh.2013.05.010 23998664

[B43] Kanaya M. Tsuda M. C. Sagoshi S. Nagata K. Morimoto C. Thu C. K. (2014). Regional difference in sex steroid action on formation of morphological sex differences in the anteroventral periventricular nucleus and principal nucleus of the bed nucleus of the stria terminalis. *PLoS One* 9:e112616. 10.1371/journal.pone.0112616 25398007 PMC4232352

[B44] Kato Y. Nakashima S. Maekawa F. Tsukahara S. (2012). Involvement of postnatal apoptosis on sex difference in number of cells generated during late fetal period in the sexually dimorphic nucleus of the preoptic area in rats. *Neurosci. Lett.* 516 290–295. 10.1016/j.neulet.2012.04.017 22521312

[B45] Kauffman A. S. (2009). Sexual differentiation and the Kiss1 system: hormonal and developmental considerations. *Peptides* 30 83–93. 10.1016/j.peptides.2008.06.014 18644414 PMC2631352

[B46] Kauffman A. S. Gottsch M. L. Roa J. Byquist A. C. Crown A. Clifton D. K. (2007). Sexual differentiation of Kiss1 gene expression in the brain of the rat. *Endocrinology* 148 1774–1783. 17204549 10.1210/en.2006-1540

[B47] Krishnan S. Intlekofer K. A. Aggison L. K. Petersen S. L. (2009). Central role of TRAF-interacting protein in a new model of brain sexual differentiation. *Proc. Natl. Acad. Sci. U.S.A.* 106 16692–16697. 10.1073/pnas.0906293106 19805359 PMC2757835

[B48] Lenz K. M. Nugent B. M. Haliyur R. McCarthy M. M. (2013). Microglia are essential to masculinization of brain and behavior. *J. Neurosci.* 33 2761–2772. 10.1523/JNEUROSCI.1268-12.2013 23407936 PMC3727162

[B49] Lenz K. M. Pickett L. A. Wright C. L. Davis K. T. Joshi A. McCarthy M. M. (2018). Mast cells in the developing brain determine adult sexual behavior. *J. Neurosci.* 38 8044–8059. 10.1523/JNEUROSCI.1176-18.2018 30093566 PMC6136154

[B50] Lenz K. M. Wright C. L. Martin R. C. McCarthy M. M. (2011). Prostaglandin E(2) regulates AMPA receptor phosphorylation and promotes membrane insertion in preoptic area neurons and glia during sexual differentiation. *PLoS One* 6:e18500. 10.1371/journal.pone.0018500 21490976 PMC3072395

[B51] Liu X. Porteous R. d’Anglemont de Tassigny X. Colledge W. H. Millar R. Petersen S. L. (2011). Frequency-dependent recruitment of fast amino acid and slow neuropeptide neurotransmitter release controls gonadotropin-releasing hormone neuron excitability. *J. Neurosci.* 31 2421–2430. 10.1523/JNEUROSCI.5759-10.2011 21325509 PMC6623676

[B52] Maejima S. Abe Y. Yamaguchi S. Musatov S. Ogawa S. Kondo Y. (2018). VGF in the medial preoptic nucleus increases sexual activity following sexual arousal induction in male rats. *Endocrinology* 159 3993–4005. 10.1210/en.2018-00804 30371765

[B53] Matsuda K. I. Mori H. Nugent B. M. Pfaff D. W. McCarthy M. M. Kawata M. (2011). Histone deacetylation during brain development is essential for permanent masculinization of sexual behavior. *Endocrinology* 152 2760–2767. 10.1210/en.2011-0193 21586557 PMC3115610

[B54] McCarthy M. M. (2019). Is sexual differentiation of brain and behavior epigenetic? *Curr. Opin. Behav. Sci.* 25 83–88. 10.1016/j.cobeha.2018.10.005 31106255 PMC6519476

[B55] McCarthy M. M. (2020). A new view of sexual differentiation of mammalian brain. *J. Comp. Physiol. A Neuroethol. Sens. NeuralBehav. Physiol.* 206 369–378. 10.1007/s00359-019-01376-8 31705197 PMC7196030

[B56] McCarthy M. M. Arnold A. P. (2011). Reframing sexual differentiation of the brain. *Nat. Neurosci.* 14 677–683. 10.1038/nn.2834 21613996 PMC3165173

[B57] Meier T. J. Ho D. Y. Park T. S. Sapolsky R. M. (1998). Gene transfer of calbindin D28k cDNA via herpes simplex virus amplicon vector decreases cytoplasmic calcium ion response and enhances neuronal survival following glutamatergic challenge but not following cyanide. *J. Neurochem.* 71 1013–1023. 10.1046/j.1471-4159.1998.71031013.x 9721726

[B58] Moe Y. Kyi-Tha-Thu C. Tanaka T. Ito H. Yahashi S. Matsuda K. I. (2016). A sexually dimorphic area of the dorsal hypothalamus in mice and common marmosets. *Endocrinology* 157 4817–4828. 10.1210/en.2016-1428 27726418

[B59] Morishita M. Koiso R. Tsukahara S. (2020). Actions of peripubertal gonadal steroids in the formation of sexually dimorphic brain regions in mice. *Endocrinology* 161:bqaa063. 10.1210/endocr/bqaa063 32303738

[B60] Morishita M. Maejima S. Tsukahara S. (2017). Gonadal hormone-dependent sexual differentiation of a female-biased sexually dimorphic cell group in the principal nucleus of the bed nucleus of the Stria Terminalis in mice. *Endocrinology* 158 3512–3525. 10.1210/en.2017-00240 28977609

[B61] Mosley M. Weathington J. Cortes L. R. Bruggeman E. Castillo-Ruiz A. Xue B. (2017). Neonatal inhibition of DNA methylation alters cell phenotype in sexually dimorphic regions of the mouse brain. *Endocrinology* 158 1838–1848. 10.1210/en.2017-00205 28398586 PMC5460944

[B62] Murray E. K. Hien A. de Vries G. J. Forger N. G. (2009). Epigenetic control of sexual differentiation of the bed nucleus of the stria terminalis. *Endocrinology* 150 4241–4247. 10.1210/en.2009-0458 19497973 PMC2736071

[B63] Nugent B. M. Wright C. L. Shetty A. C. Hodes G. E. Lenz K. M. Mahurkar A. (2015). Brain feminization requires active repression of masculinization via DNA methylation. *Nat. Neurosci.* 18 690–697. 10.1038/nn.3988 25821913 PMC4519828

[B64] Orikasa C. Sakuma Y. (2010). Estrogen configures sexual dimorphism in the preoptic area of C57BL/6J and ddN strains of mice. *J. Comp. Neurol.* 518 3618–3629. 10.1002/cne.22419 20593361

[B65] Ottem E. N. Godwin J. G. Krishnan S. Petersen S. L. (2004). Dual-phenotype GABA/glutamate neurons in adult preoptic area: sexual dimorphism and function. *J. Neurosci.* 24 8097–8105. 10.1523/JNEUROSCI.2267-04.2004 15371511 PMC6729791

[B66] Patchev A. V. Gotz F. Rohde W. (2004). Differential role of estrogen receptor isoforms in sex-specific brain organization. *FASEB J.* 18 1568–1570. 10.1096/fj.04-1959fje 15289439

[B67] Sano K. Nakata M. Musatov S. Morishita M. Sakamoto T. Tsukahara S. (2016). Pubertal activation of estrogen receptor alpha in the medial amygdala is essential for the full expression of male social behavior in mice. *Proc. Natl. Acad. Sci. U.S.A.* 113 7632–7637. 10.1073/pnas.1524907113 27325769 PMC4941494

[B68] Schmidt H. (2012). Three functional facets of calbindin D-28k. *Front. Mol. Neurosci.* 5:25. 10.3389/fnmol.2012.00025 22435048 PMC3304297

[B69] Schulz K. M. Sisk C. L. (2016). The organizing actions of adolescent gonadal steroid hormones on brain and behavioral development. *Neurosci. Biobehav. Rev.* 70 148–158. 10.1016/j.neubiorev.2016.07.036 27497718 PMC5074860

[B70] Scott N. Prigge M. Yizhar O. Kimchi T. (2015). A sexually dimorphic hypothalamic circuit controls maternal care and oxytocin secretion. *Nature* 525 519–522. 10.1038/nature15378 26375004

[B71] Semaan S. J. Murray E. K. Poling M. C. Dhamija S. Forger N. G. Kauffman A. S. (2010). BAX-dependent and BAX-independent regulation of Kiss1 neuron development in mice. *Endocrinology* 151 5807–5817. 10.1210/en.2010-0783 20926580 PMC2999490

[B72] Sickel M. J. McCarthy M. M. (2000). Calbindin-D28k immunoreactivity is a marker for a subdivision of the sexually dimorphic nucleus of the preoptic area of the rat: developmental profile and gonadal steroid modulation. *J. Neuroendocrinol.* 12 397–402. 10.1046/j.1365-2826.2000.00474.x 10792577

[B73] Simerly R. B. Swanson L. W. Gorski R. A. (1985a). The distribution of monoaminergic cells and fibers in a periventricular preoptic nucleus involved in the control of gonadotropin release: immunohistochemical evidence for a dopaminergic sexual dimorphism. *Brain Res.* 330 55–64. 10.1016/0006-8993(85)90007-1 2859086

[B74] Simerly R. B. Swanson L. W. Handa R. J. Gorski R. A. (1985b). Influence of perinatal androgen on the sexually dimorphic distribution of tyrosine hydroxylase-immunoreactive cells and fibers in the anteroventral periventricular nucleus of the rat. *Neuroendocrinology* 40 501–510. 10.1159/000124122 2861581

[B75] Simerly R. B. Zee M. C. Pendleton J. W. Lubahn D. B. Korach K. S. (1997). Estrogen receptor-dependent sexual differentiation of dopaminergic neurons in the preoptic region of the mouse. *Proc. Natl. Acad. Sci. U.S.A.* 94 14077–14082. 10.1073/pnas.94.25.14077 9391155 PMC28435

[B76] Sumida H. Nishizuka M. Kano Y. Arai Y. (1993). Sex differences in the anteroventral periventricular nucleus of the preoptic area and in the related effects of androgen in prenatal rats. *Neurosci. Lett.* 151 41–44. 10.1016/0304-3940(93)90040-r 8469435

[B77] Tsukahara S. Hojo R. Kuroda Y. Fujimaki H. (2008). Estrogen modulates Bcl-2 family protein expression in the sexually dimorphic nucleus of the preoptic area of postnatal rats. *Neurosci. Lett.* 432 58–63. 10.1016/j.neulet.2007.12.006 18164816

[B78] Tsukahara S. Kakeyama M. Toyofuku Y. (2006). Sex differences in the level of Bcl-2 family proteins and caspase-3 activation in the sexually dimorphic nuclei of the preoptic area in postnatal rats. *J. Neurobiol.* 66 1411–1419. 10.1002/neu.20276 17013925

[B79] Tsukahara S. Tsuda M. C. Kurihara R. Kato Y. Kuroda Y. Nakata M. (2011). Effects of aromatase or estrogen receptor gene deletion on masculinization of the principal nucleus of the bed nucleus of the stria terminalis of mice. *Neuroendocrinology* 94 137–147. 10.1159/000327541 21525731

[B80] Tsukamura H. Homma T. Tomikawa J. Uenoyama Y. Maeda K. (2010). Sexual differentiation of kisspeptin neurons responsible for sex difference in gonadotropin release in rats. *Ann. N. Y. Acad. Sci.* 1200 95–103. 10.1111/j.1749-6632.2010.05645.x 20633137

[B81] Waters E. M. Simerly R. B. (2009). Estrogen induces caspase-dependent cell death during hypothalamic development. *J. Neurosci.* 29 9714–9718. 10.1523/JNEUROSCI.0135-09.2009 19657024 PMC6428191

[B82] Wittmann W. McLennan I. S. (2013a). Anti-Mullerian hormone may regulate the number of calbindin-positive neurons in the sexually dimorphic nucleus of the preoptic area of male mice. *Biol. Sex Differ.* 4 18. 10.1186/2042-6410-4-18 24119315 PMC3852321

[B83] Wittmann W. McLennan I. S. (2013b). The bed nucleus of the stria terminalis has developmental and adult forms in mice, with the male bias in the developmental form being dependent on testicular AMH. *Horm. Behav.* 64 605–610. 10.1016/j.yhbeh.2013.08.017 24012942

[B84] Woodson J. C. Balleine B. W. Gorski R. A. (2002). Sexual experience interacts with steroid exposure to shape the partner preferences of rats. *Horm. Behav.* 42 148–157. 10.1006/hbeh.2002.1816 12367568

[B85] Wright C. L. Burks S. R. McCarthy M. M. (2008). Identification of prostaglandin E2 receptors mediating perinatal masculinization of adult sex behavior and neuroanatomical correlates. *Dev. Neurobiol.* 68 1406–1419. 10.1002/dneu.20665 18726914 PMC2725403

[B86] Wright C. L. McCarthy M. M. (2009). Prostaglandin E2-induced masculinization of brain and behavior requires protein kinase A, AMPA/kainate, and metabotropic glutamate receptor signaling. *J. Neurosci.* 29 13274–13282. 10.1523/JNEUROSCI.3603-09.2009 19846715 PMC3568388

[B87] Yamaguchi S. Abe Y. Maejima S. Tsukahara S. (2018). Sexual experience reduces neuronal activity in the central part of the medial preoptic nucleus in male rats during sexual behavior. *Neurosci. Lett.* 685 155–159. 10.1016/j.neulet.2018.08.037 30170041

[B88] Yoshida M. Yuri K. Kizaki Z. Sawada T. Kawata M. (2000). The distributions of apoptotic cells in the medial preoptic areas of male and female neonatal rats. *Neurosci. Res.* 36 1–7. 10.1016/s0168-0102(99)00100-5 10678526

[B89] Zup S. L. Carrier H. Waters E. M. Tabor A. Bengston L. Rosen G. J. (2003). Overexpression of bcl-2 reduces sex differences in neuron number in the brain and spinal cord. *J. Neurosci.* 23 2357–2362. 10.1523/jneurosci.23-06-02357.2003 12657695 PMC6742046

